# Fungi and cancer: unveiling the complex role of fungal infections in tumor biology and therapeutic resistance

**DOI:** 10.3389/fcimb.2025.1596688

**Published:** 2025-06-10

**Authors:** Wanli Zhang, He Zhang, Yiru Gao, Jianjun Lei, Chenhao Suo

**Affiliations:** ^1^ College of Life and Health Science, Northeastern University, Shenyang, Liaoning, China; ^2^ Department of Laboratory Animal Center, General Hospital of Northern Theater Command, Shenyang, Liaoning, China

**Keywords:** cancer, tumor microenvironment, immunity, fungal derived metabolites, antifungal therapy fungi, fungal-derived metabolites, antifungal therapy

## Abstract

Cancer remains one of the most significant causes of mortality across the world. Despite remarkable advancements made in early detection, therapeutic strategies, and the advent of immunotherapy in recent years, numerous challenges continue to hinder optimal outcomes. The development and progression of cancer are driven not only by genetic and epigenetic alterations within tumor cells but also by dynamic interactions occurring with the surrounding tumor microenvironment (TME). It is a highly complex milieu composed of tumor cells, non-tumor stromal cells, extracellular matrix components, immune cells, blood vessels, and diverse signaling molecules. Emerging evidence underscores the pivotal role of fungi in influencing cancer biology, including initiation, progression, immune evasion, and the modulation of TME. Fungi, which are omnipresent microorganisms, have traditionally been considered opportunistic pathogens. However, recent research highlights their broader impact on host immunity and their potential contributions to cancer pathogenesis. For instance, in patients with cancer, fungal infections not only exacerbate clinical complications but also create conditions conducive to tumor growth, metastasis, and immune escape by altering the immune microenvironment. In addition, fungal-derived metabolites and their interactions with host immune pathways can significantly modulate the efficacy of immunotherapies. These findings have spurred interest in exploring antifungal strategies as adjunctive approaches in cancer management, positioning antifungal therapy as a burgeoning area of oncological research. This review provides an in-depth exploration of the complex interplay between fungi and cancer. It examines the multifaceted role of fungal infections in tumor biology, the mechanisms through which fungi reshape the TME through immune modulation and their influence on immune-evasion strategies and therapeutic resistance. Furthermore, the potential for integrating antifungal therapies into comprehensive cancer treatment regimens has been highlighted, offering insights into novel avenues for improving patient outcomes.

## 1 Introduction

**Figure 1:**
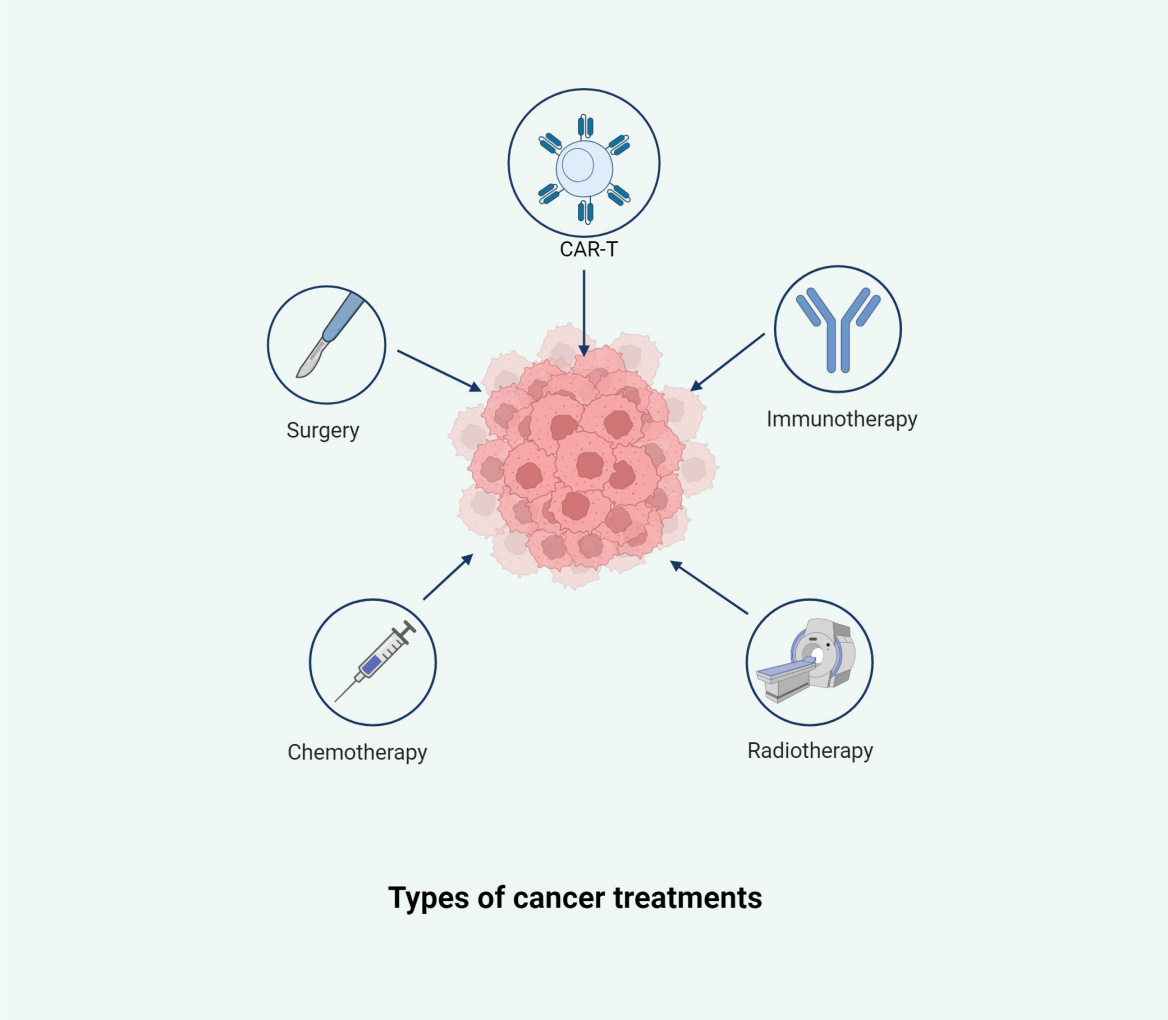
This illustration summarizes the primary therapeutic strategies employed in cancer management, including surgery, chemotherapy, radiotherapy, immunotherapy, and chimeric antigen receptor T-cell (CAR-T) therapy. Each modality targets the tumor from a distinct angle, aiming to remove, destroy, or modulate malignant cells and the tumor microenvironment. These approaches are often used in combination to enhance efficacy and reduce the risk of recurrence.

Cancer is one of the most significant causes of mortality globally (Mullard, 2020). According to the World Health Organization, nearly 10 million people succumbed to cancer in 2020 alone (Mullard, 2020) (Liu et al., 2023a). In the relentless effort to combat this disease, research and advancements in cancer treatment have continued unabated (Dolgin, 2021; Gilbertson, 2011; Kroemer and Pouyssegur, 2008). Cancer management is a multifaceted and highly individualized process, influenced by factors such as the cancer type, stage, patient health, and the molecular characteristics of the tumor (Duggan et al., 2017; Hui and Bruera, 2020; Jost and Roila, 2009; Samuel et al., 2014). Traditional treatment modalities, including surgery, radiotherapy, and chemotherapy, remain foundational (Allen et al., 2017) (**Figure 1**). However, the emergence of cancer immunotherapy in recent years represents a transformative breakthrough in oncology (Borgers et al., 2021; Derynck et al., 2021; Morrison et al., 2018; Szeto and Finley, 2019).

Immunotherapy functions by enhancing or modulating the patient’s immune system to recognize and destroy cancer cells (Brandenburg et al., 2024). A prominent example of this is immune checkpoint inhibitors (Abril-Rodriguez and Ribas, 2017), which block inhibitory signals that suppress immune responses, thereby activating the immune system to target malignant cells (Abril-Rodriguez and Ribas, 2017; Curry and Lim, 2015). This approach has demonstrated remarkable efficacy in treating various types of cancers, including melanoma and non-small-cell lung cancer (Arbour and Riely, 2019; Derosa et al., 2018; McGranahan et al., 2016).

Another innovative therapy is chimeric antigen receptor T-cell therapy, wherein patient’s T-cells are genetically engineered to identify and attack cancer cells (Gumber and Wang, 2022; Liu et al., 2022b). While chimeric antigen receptor T-therapy has demonstrated significant success in hematological malignancies such as leukemia and lymphoma (Uscanga-Palomeque et al., 2023), its application to solid tumors presents substantial challenges that remain the focus of ongoing research (Chohan et al., 2023; Ma et al., 2019; Uscanga-Palomeque et al., 2023; Zhang et al., 2023, 2022).

Cancer vaccines represent a preventative approach, with notable examples including the human papillomavirus vaccine and the hepatitis B vaccine (Liu et al., 2023c; Saxena et al., 2021), both of which effectively prevent certain virus-associated cancers (Liu et al., 2024e). However, the development of therapeutic cancer vaccines is still in its early stages (Liu et al., 2024e; Lopes et al., 2019).

Targeted therapy is another promising strategy in cancer treatment (Chang et al., 2021). By aiming at specific genetic mutations or molecular markers unique to cancer cells (Chang et al., 2021), it disrupts tumor growth with greater precision relative to that of the traditional chemotherapy (Crooke et al., 2018; Liu and Wang, 2024), thereby sparing normal cells and minimizing the inevitable collateral damage (Crooke et al., 2018). Several targeted agents have been successfully integrated into clinical practice (Liu and Wang, 2024; Mancarella et al., 2023; Wang et al., 2020), such as small-molecule epidermal growth factor receptor inhibitors (e.g., gefitinib and erlotinib) (Damaraju et al., 2014; Kohsaka et al., 2017; Yang et al., 2017) and human epidermal growth factor receptor 2-targeted drugs (e.g., trastuzumab) (Cameron et al., 2017; Hurvitz et al., 2023). Monoclonal antibodies, including trastuzumab and bevacizumab (Limousin et al., 2023; Pierga et al., 2012; Wahid et al., 2016), bind to surface antigens on cancer cells, leading to their destruction or inhibiting their proliferation, yielding significant clinical benefits (Singh et al., 2024).

The introduction of immune checkpoint inhibitors has revolutionized cancer therapy, shifting scientific attention to the critical role of immune evasion within the tumor microenvironment (TME) (Galassi et al., 2024). Therefore, understanding and manipulating the TME to overcome immunosuppressive mechanisms and enhance immune-mediated tumor destruction is now a key research priority, driving innovation in the next-generation immunotherapies (Barkley et al., 2022; Galassi et al., 2024; Tang et al., 2021; Zhang et al., 2024).

## 2 TME and microbial relationships

The relationship between the TME and the microbiota has emerged as a dynamic and growing field in the cancer research domain (Lam et al., 2021; Lu et al., 2022). Historically, cancer studies have primarily focused on tumor cells, the immune system, and genetic mutations, often overlooking the role of microbiota (Goenka et al., 2023; Liu et al., 2024b). However, advancements in microbiomics and immunology have increasingly highlighted the profound influence of the microbiota (Liu et al., 2024b), particularly bacteria, within the tumor environment on processes such as tumor initiation, progression, metastasis, immune evasion, and therapeutic response (Guillot et al., 2023; Jiang et al., 2023; Liu et al., 2024b; You et al., 2024).

While bacteria have garnered substantial attention over the past few decades for their role in cancer (Souza et al., 2023), fungi—a significant component of the microbiota—have been comparatively understudied (Gilbertson, 2011; Kroemer and Pouyssegur, 2008). Fungi encompass a diverse range of organisms, including clinically relevant pathogens such as *Aspergillus*, *Candida*, *Cryptococcus*, and *Pneumocystis* (Strickland and Shi, 2021). In nature, fungi have become indispensable for ecological balance (Dean et al., 2012; Strickland and Shi, 2021), contributing to organic matter decomposition, nutrient cycling, and forming symbiotic relationships with other organisms (Biedermann and Vega, 2020; Jia and Chen, 2025).

Despite their larger size compared to bacteria, fungi account for a minor proportion of the gut microbiota in terms of abundance but may have disproportionate effects on host immunity and metabolism, fungi represent only approximately 0.6% of total microbial DNA (Chen et al., 2017; Takeuchi et al., 2024), which has led to the underestimation of their potential pathogenicity. Fungal infections account for more than 1.5 million deaths annually and have significant implications on the host immune system as well as the overall microbiota composition. Recent research has confirmed that fungi play a critical role in shaping the TME, thereby influencing cancer development and progression (Wang et al., 2024c).

Although fungi comprise only 0.01–2% of the gut microbiota, their functional impact on health and disease far exceeds this proportion (Cheng et al., 2024; Dart, 2019; Galloway-Peña et al., 2024; Saftien et al., 2023). Their interactions with bacteria and the host immune system are highly complex (Belkaid and Hand, 2014; Mazmanian et al., 2005; Sepich-Poore et al., 2021). In addition, owing to their larger cellular size, the total biomass of symbiotic fungi possibly surpassed that of bacteria (How et al., 2022; Saftien et al., 2023). Traditionally, fungi have been studied primarily in the context of infectious diseases (Fernandes and Carter, 2020; Strickland and Shi, 2021; Wang et al., 2024a), while their role in symbiosis remains underexplored (Dohlman et al., 2022; Narunsky-Haziza et al., 2022). Moreover, certain symbiotic fungi exhibit opportunistic behavior, adding complexity to their functional roles (Dohlman et al., 2022; Narunsky-Haziza et al., 2022). Research efforts are further complicated by the high individual and temporal variability in microbiota composition, which often exceeds that observed in bacterial communities (Lin et al., 2022; Liu et al., 2022a).

Most studies have indicated that the *Ascomycota* and *Basidiomycota* phyla dominate fungal populations across various body sites (Angelova et al., 2023; Guarro et al., 1999; Sandargo et al., 2019), with the gut representing the most extensively studied ecological niche owing to its dense microbial ecosystem (Saftien et al., 2023).

Fungi exist in diverse forms, including yeasts and hyphae (Witchley et al., 2019). The ability of yeasts to convert into hyphae is a key pathogenic trait of fungi like *Candida albicans* (Honorato et al., 2022; Kiss et al., 2019; Witchley et al., 2019). Yeast cells are generally more resistant to macrophage killing and immune responses when compared to their hyphal forms (Witchley et al., 2019). The transition from the yeast form to the hyphae form is typically influenced by environmental factors such as pH, CO_2_ levels, anaerobic conditions, and temperature (Honorato et al., 2022; Lu et al., 2014; Sudbery, 2011). Fungi inhabit multiple ecological niches in the human body, including the gastrointestinal tract and the surfaces of other mucosal membranes (Lohse et al., 2018; Soll, 2024). The relationship between fungi and human health is deeply interconnected (Lohse et al., 2018).

Fungal infections can be classified into two categories: superficial and systemic (Brown et al., 2012).

Superficial infections: These include infections of the skin, nails, and mucous membranes (Mayer et al., 2013; Otašević and Hay, 2023). The most common examples are *Candida* infections (e.g., oral thrush and vaginal candidiasis) and dermatophyte infections (e.g., tinea capitis, tinea corporis, and tinea cruris) (Mayer et al., 2013).

Systemic infections: These infections occur when the immune system is compromised, allowing fungal infections to spread to the internal organs (Bing et al., 2024). Common examples include pulmonary infections (e.g., aspergillosis and cryptococcosis) and bloodstream infections (e.g., *Candida* bloodstream infections) (Esher Righi et al., 2023). These infections tend to be more severe and are especially threatening to immunocompromised patients (Esher Righi et al., 2023; Morales-López et al., 2017; Park et al., 2024).

In recent years, the incidence of fungal infections has steadily increased owing to the widespread use of antibiotics and immunosuppressive drugs (Parsons and Diekema, 2023; Spallone and Schwartz, 2021). This rise is particularly noticeable among cancer patients, organ transplant recipients, and individuals with HIV/AIDS, in whom fungal infections pose a serious complication (Benitez and Carver, 2019; Brüggemann et al., 2022; Gøtzsche and Johansen, 2014; Pagano and Caira, 2014). Some of the most commonly used antifungal drugs are as follows:

Fluconazole and itraconazole (broad-spectrum antifungal agents mainly used for treating systemic fungal infections).

Amphotericin B (a broad-spectrum antifungal drug that is often used for treating severe fungal infections).

Voriconazole (primarily used for treating invasive fungal infections) (Arendrup et al., 2020; Chen and Sorrell, 2007).

However, the increasing use of antifungal treatments has seen a parallel rise in the corresponding antifungal resistance (Kozubowski and Berman, 2025; Lockhart et al., 2023), especially with *Candida* species and *Aspergillus* species, which are developing resistance to standard antifungal medications (Carolus et al., 2021; Gregor et al., 2023; Lee et al., 2021). This aspect is driven by research efforts to develop new antifungal drugs (Nicola et al., 2019).

In the early 20^th^ century, scientists began recording a co-occurrence between certain fungal infections and cancer in patients (Liu et al., 2023a). However, most of these studies were descriptive and did not explore the potential role of fungi in cancer development (Wu et al., 2024a). In recent years, the rapid advancement of omics research has facilitated the unraveling of the relationship between fungi and cancer, revealing a deeper and more complex connection.

## 3 Literature search and inclusion criteria

To ensure a comprehensive overview of the topic, we conducted a literature search using PubMed, Scopus, and Web of Science databases up to February 2025. The keywords included “fungi and cancer,” “mycobiome and tumor,” “fungal metabolites and carcinogenesis,” “antifungal therapy and oncology,” among others. We included English-language peer-reviewed articles focusing on experimental models, clinical studies, or mechanistic insights related to fungi and cancer. Articles were screened based on relevance, and duplicates were removed.

### 3.1 Fungi as diagnostic biomarkers

**Figure 2:**
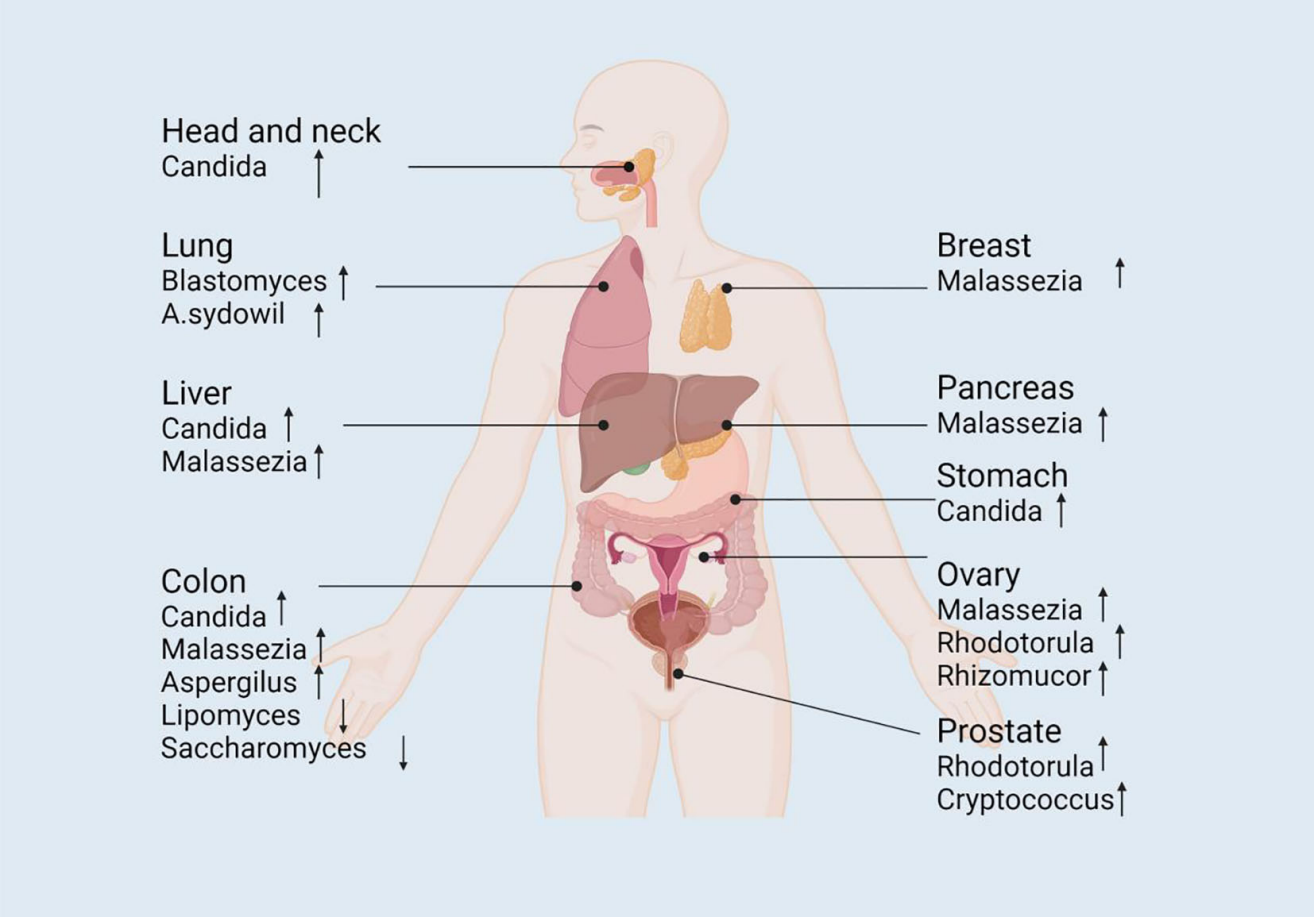
The alteration of mycobiome in abundance across different tumor sites. The composition of fungal mycobiome is altered in different body sites (e.g., colorectum, pancreas, stomach, liver, head and neck, lung, and breast) that are associated with tumorigenesis, serving as potential diagnostic or prognostic biomarkers to promote the study of the complicated mechanistic investigation of fungal involvement in carcinogenesis. ↓decrease; ↑increase. Pathways with dashed arrows represent hypothetical interactions yet to be validated in clinical studies (Dohlman et al., 2022; Su et al., 2024).

Research on fungi as tumor biomarkers has emerged as a new field in recent years. The conventional tumor biomarkers are mainly based on tumor cells or their metabolic products (Dohlman et al., 2022). However, past studies have suggested that certain fungi and their metabolites display distinct changes in cancer patients, implicating their potential applications in the early diagnosis, prediction, and treatment of tumors (Dohlman et al., 2022; Lin et al., 2022; Liu et al., 2022a; Su et al., 2024) (**Figure 2**).

#### 3.1.1 Diagnostic potential of fungi as biomarkers in gastrointestinal tumors

The gastrointestinal tract is the area with the highest prevalence of fungi in the human body (Dohlman et al., 2022; Wu et al., 2024a). Researchers have identified the presence of fungi in gastrointestinal tumors as well as discovered a close association between fungi and cancer development (Dohlman et al., 2022; Lin et al., 2022; Liu et al., 2022a; Su et al., 2024).

Globally, colorectal cancer (CRC) is one of the most common causes of death, with continuously rising incidence rates, accounting for approximately 900,000 deaths annually (Dekker et al., 2019). Past studies have reported that the occurrence of CRC correlates with fungal abundance relative to that in healthy controls (Bai et al., 2022; Schürch et al., 2020). These alterations include an enrichment of the *Basidiomycota*/*Ascomycota* ratio (Sokol et al., 2017), an abundance of *Malasseziomycetes*, and a depletion of *Saccharomycetes* and *Pneumocystidomycetes* proportion (Coker et al., 2019). Several studies have observed an increased *Basidiomycota*/*Ascomycota* ratio in colorectal cancer patients; however, most of these studies involve small cohorts and observational designs, and thus the predictive value remains speculative and requires further validation in larger, well-controlled studies. Furthermore, the population of specific fungal species such as *Lipomyces starkeyi* and *Saccharomyces cerevisiae* are reduced, while those of others like *Malassezia globosa* and *Aspergillus flavus* are enriched in CRC patients (Coker et al., 2019). Moreover, past research has revealed that a combination of fungal and bacterial biomarkers was more accurate in distinguishing CRC patients from healthy individuals when compared to using only bacterial species (Lin et al., 2022; Liu et al., 2022a).

In patients with liver and gastric cancers (GC), a decrease in alpha diversity and an increase in the number of opportunistic fungi (such as *Malassezia* and *Candida*) have been detected (Zhong et al., 2021). For instance, Dohlman reported that *C. albicans* mediates GC by reducing the diversity and richness of gastric fungi, thereby promoting the pathogenesis of GC (Dohlman et al., 2022). A similar phenomenon was observed in adenomas, wherein fungal diversity was reduced in comparison to healthy tissues (Luan et al., 2015). Furthermore, Aykut et al. reported microbial dysbiosis in the tumors of pancreatic and oral cancer patients in both mouse and human studies (Aykut et al., 2019).

#### 3.1.2 Diagnostic potential in non-gastrointestinal cancers

A similar phenomenon has been observed in non-gastrointestinal cancers, demonstrating the significant potential of fungi as tumor biomarkers.

Through the Cancer Genome Atlas cohort, detected an enrichment of *Blastomyces dermitidis/gilchristii* in cancer patients. Notably, in the Weizmann cohort, smokers displayed a higher abundance of *Aspergillus* and *Agaricus* in their tumors compared to non-smokers with lung cancer (Narunsky-Haziza et al., 2022). In breast cancer, *Malassezia* was found to be significantly enriched, while *Aspergillus* and *Malassezia* were found to form a hub for fungal-bacterial co-occurrence (Dohlman et al., 2022) (Narunsky-Haziza et al., 2022). When compared to patients with cirrhosis, those with hepatocellular carcinoma (HCC) exhibited significantly reduced gut microbiome diversity, but an increase in *C. albicans* abundance (Liu et al., 2022c).

In 2022, a collaborative study between the Weizmann Institute of Science (Israel) and the University of California, San Diego (USA) comprehensively characterized cancer microbiota in 17,401 patients across four independent cohorts with 35 cancer types. The study reported a low abundance of fungal DNA and cells in several major human cancers when compared with fungal communities with matching bacterial communities and immune profiles; this study also explored the role of fungi in prognosis and diagnosis. The results of this past study provided new insights into cancer detection and treatment (Narunsky-Haziza et al., 2022).

The research on fungi as tumor biomarkers is progressing rapidly, especially in the areas of specific metabolic products (Shuai et al., 2022; Vitali et al., 2022), DNA/RNA detection, and microbiome structure analysis (Liao et al., 2023). Although multi-omics studies offer integrative insights, most suffer from small sample sizes, lack of replication, and inconsistent bioinformatics pipelines, limiting their generalizability. In the future, by integrating multi-omics technologies and efficient detection methods, fungal biomarkers are expected to play a crucial role in early cancer diagnosis, treatment monitoring, and prognosis assessment (Galloway-Peña et al., 2024; Liu et al., 2022a; Saftien et al., 2023). In addition, investigating the interactions and regulatory mechanisms between fungi and the TME is expected to further promote the development of precision medicine in this are (Hiam-Galvez et al., 2021; Ruffin et al., 2023).

### 3.2 Fungi Affecting TME through immune modulation

**Figure 3:**
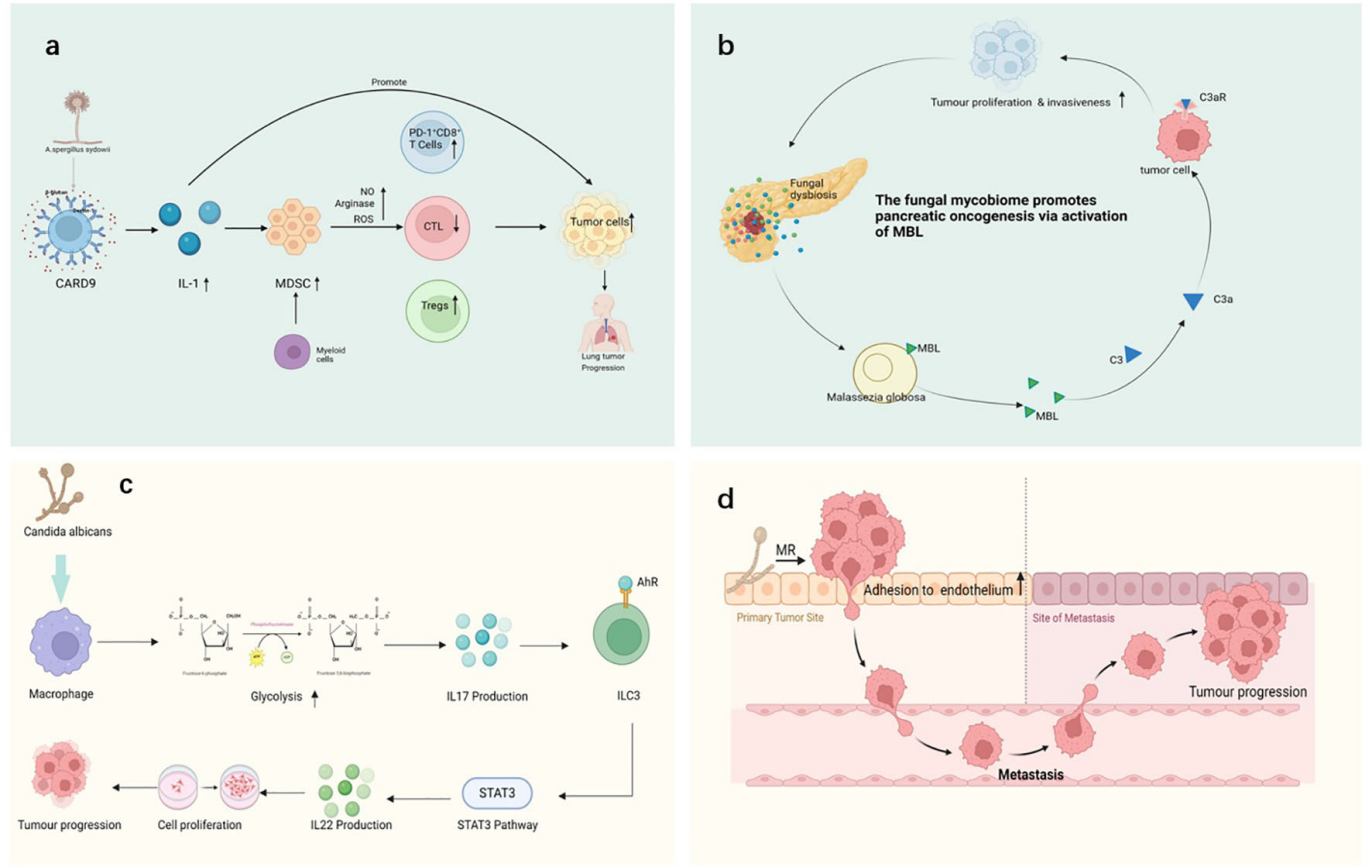
Various mechanisms through which fungi interact with cancer, including: **(a)** *A sydowii* activates immune responses through the CARD9 pathway, promoting the upregulation of IL-1 and myeloid-derived suppressor cells (MDSCs). By inducing the production of nitric oxide (NO), arginase, and reactive oxygen species (ROS), it impairs the effect of cytotoxic T-lymphocytes (CTLs) and increases the proportion of PD-1+ CD8+ T-cells. These immune responses may contribute to tumor progression, causing the proliferation of lung cancer cells. **(b)** The activation of *Malassezia globosa* triggers the mannose-binding lectin (MBL) pathway, the MBL (mannose-binding lectin) pathway initiates complement activation via MASP-1 and MASP-2, which cleave C4 and C2 to generate C3 convertase, leading to downstream immune signaling, promoting the development of pancreatic cancer. This process involves tumor proliferation, invasiveness, and immune modulation. **(c)** In murine models, C albicans infection has been shown to enhance IL-17 production via macrophage glycolytic reprogramming, subsequently activating ILC3 cells and promoting IL-22 secretion through the VEGF 3 pathway. While this cascade has been linked to increased tumor proliferation in experimental systems, its clinical relevance remains debated, and contradictory data suggest that IL-17 may have dual roles depending on cancer type and immune context (Aggor et al., 2020; X. Wang et al., 2023b). **(d)** Fungi enhance tumor cell adhesion to endothelial cells through interactions with tumor cell surface mannose receptors (MR), thereby facilitating the metastasis of cancer cells. This mechanism allows tumor cells to migrate from the primary site to metastatic sites, driving cancer progression. Pathways with dashed arrows represent hypothetical interactions yet to be validated in clinical studies (Heung et al., 2023; Riquelme and McAllister, 2021; Sheng et al., 2024; Soerens et al., 2023).

The impact of fungi on the TME is a complex and increasingly focused area of research (Jiang et al., 2022b). Fungi not only play important roles in human health but also influence the occurrence, development, and response to treatments through various mechanisms (Saftien et al., 2023). Fungi can alter the TME both directly and indirectly, thereby affecting processes such as tumor cell proliferation, immune evasion, and metastasis (Blake et al., 2024; Cao et al., 2024; Saftien et al., 2023; Wang et al., 2024c) (**Figure 3**).

Fungi can significantly alter the TME by regulating immune cell functions, thereby influencing tumor growth, immune evasion, and response to treatment (Blake et al., 2024; Saftien et al., 2023; Wang et al., 2024c) (**Figures 3A, B**). Fungal infections not only directly affect the host immune system through their pathogenicity but also regulate immune cell functions via a range of mechanisms, which results in immune suppression, immune evasion, and a chronic inflammatory environment that favors tumor growth and metastasis (Heung et al., 2023; Riquelme and McAllister, 2021; Sheng et al., 2024; Soerens et al., 2023). Several of the key pathways through which fungi modulate immune cell functions and alter the TME are discussed below:

#### 3.2.1 Fungi modulating dendritic cells to alter the TME

DCs serve as a bridge between the innate and adaptive immune systems, initiating T-cell immune responses by phagocytosing and presenting antigens (Palucka and Banchereau, 2012). Fungi interact with DCs through their cell wall components (such as β-glucans and chitin) and other molecules (e.g., lipids and carbohydrates), binding to pattern-recognition receptors (such as Dectin-1 and TLR2) and affecting the function of DCs (Chamilos et al., 2010; Karnam et al., 2021; Koning and Mebius, 2016).

##### 3.2.1.1 Induction of immune tolerance

After activation, DCs secrete pro-inflammatory or anti-inflammatory cytokines, which determines the type of immune response (Nelson et al., 2020; Ramirez-Ortiz and Means, 2012). However, fungal infections, particularly chronic infections, often result in alterations of the DC function, converting them into immune-suppressive types (Anderson et al., 2021; Gringhuis et al., 2022). Chronic fungal infections may induce immune tolerance in DCs when initiating adaptive immune responses by secreting immunosuppressive factors such as IL-10 and TGF-β (Iberg et al., 2017). This aspect suppresses the activity of effector T-cells, leading to immune evasion in the TME(Y. Guo et al., 2020; Iberg et al., 2017).

##### 3.2.1.2 Polarization of DCs

For instance, fungi such as *C. albicans* can activate the Th17 response of DCs via β-glucans (Li et al., 2022b) (**Figure 3C**). This response leads to the secretion of large amounts of IL-17, which promotes chronic inflammation and increases the accumulation of immunosuppressive cells (e.g., Tregs), thereby altering the immune characteristics of the TME (Ramirez-Garcia et al., 2016).

#### 3.2.2 Macrophage polarization and its impact on the TME

Macrophages are important effector cells of the immune system, capable of regulating immune responses through pathogen phagocytosis and cytokine secretion (Varol et al., 2015; Wynn and Vannella, 2016). Fungal infections activate different functional states of macrophages through their surface molecules (such as β-glucans), which, especially, affect their polarization (Casadevall, 2022; Gilbert et al., 2014).

##### 3.2.2.1 Macrophages and immune suppression

M1 macrophages are pro-inflammatory and can enhance anti-tumor immune responses by secreting cytokines (such as TNF-α and IL-12) (Li et al., 2023b; Pu and Ji, 2022). Fungal infections may initially activate M1 macrophages, but, as the infection progresses, macrophages tend to polarize into M2 macrophages (Chen et al., 2024a, 2023a; Kaplanov et al., 2019). M2 macrophages secrete immunosuppressive factors (such as IL-10 and TGF-β), promoting tumor immune evasion and angiogenesis, thereby providing a favorable environment for tumor cell growth and metastasis (Chen et al., 2023a; Wang et al., 2023b; Zhou et al., 2019).

##### 3.2.2.2 Impact of fungal infection on macrophage polarization

For example, *Aspergillus* infections can lead to M2 macrophage polarization(J. J. Chen et al., 2020; Guibo et al., 2024), thereby inhibiting effector immune responses and enhancing the immunosuppressive environment in the TME (Casadevall, 2022; Sprenger et al., 2018), which, in turn, supports tumor growth and metastasis (Ma et al., 2024; Matusiak et al., 2024; Narunsky-Haziza et al., 2022) (**Figure 3D**).

#### 3.2.3 Fungi modulating T-cell function to alter the TME

T-cells play a central role in the tumor immune responses, and fungi can modulate T-cell functions through direct or indirect mechanisms, thereby influencing the TME (Chapman et al., 2020; Song et al., 2023; Zhou et al., 2024a).

##### 3.2.3.1 Treg cell accumulation and immune suppression

Regulatory T-cells (Tregs) play a critical role in immune tolerance and evasion (Savage et al., 2020; Wing et al., 2019). Fungi activate DCs and macrophages to promote the proliferation and accumulation of Tregs (Atarashi et al., 2011; Sui et al., 2020), which secrete immunosuppressive factors such as IL-10 and TGF-β, inhibiting effector T-cell function and leading to immune evasion (Arpaia et al., 2013; Atarashi et al., 2013; Furusawa et al., 2013). For instance, *Aspergillus* infection triggers an increase in the number of Tregs (Yan et al., 2021), thereby enhancing the tumor’s immune evasion mechanisms and inhibiting anti-tumor immune responses (Hezaveh et al., 2022; Kumagai et al., 2024; Moreno Ayala et al., 2023).

##### 3.2.3.2 Role of Th17 cells and IL-17

Fungal infections often promote the activation of Th17 cells, which increases the production of IL-17 (Cifaldi et al., 2022; Mills, 2023). IL-17 promotes chronic inflammation in the TME and, in some cases, enhances immune suppression (Seif et al., 2023; Sun et al., 2022). IL-17 not only activates immune cells but also induces local immune tolerance, thereby increasing the accumulation of Tregs and further inhibiting anti-tumor immune responses (Fidelle et al., 2023; Lee et al., 2023).

#### 3.2.4 Accumulation of immunosuppressive cells to alter the TME

Fungi alter the immune status of the TME by affecting the function of immunosuppressive cells (such as M2 macrophages, Tregs, and myeloid-derived suppressor cells [MDSCs]), which promotes tumor growth and metastasis (Lyu et al., 2022; Xu et al., 2018; Zhao et al., 2018).

##### 3.2.4.1 Role of MDSCs

MDSCs are immune-suppressive cell populations in cancer and chronic infection (Nakamura and Smyth, 2020; Schneider et al., 2022; Yang et al., 2022b). They suppress the function of effector T-cells by secreting immunosuppressive factors (such as TGF-β and IL-10), which, in turn, promotes tumor immune evasion (Cervantes-Villagrana et al., 2020; Kuang et al., 2023; Yi et al., 2023). Fungal infections (e.g., *Cryptococcus* infection) can enhance the generation and function of MDSCs (Li et al., 2022c), leading to the generation of an immunosuppressive environment in the TME and the reduction in the effectiveness of anti-tumor immune responses (Li et al., 2022c).

##### 3.2.4.2 Immune evasion and immune tolerance

Fungi activate immunosuppressive cells such as Tregs, M2 macrophages, and MDSCs, which facilitate the immune evasion of tumors (Marcos et al., 2016; Pais et al., 2016). For example, chronic *Candida* infection can secrete immunosuppressive factors such as IL-10 (Candon et al., 2020), thereby inhibiting the activity of effector immune cells and supporting tumor cell growth and immune evasion (Candon et al., 2020; MaChado and Torres, 2018; Szabo et al., 2023).

#### 3.2.5 Fungi’s impact on TME via immune evasion mechanisms

Fungal infections can induce immune evasion through various mechanisms, thereby supporting tumors (Cheng et al., 2024). The key mechanisms of immune evasion are discussed below (Cheng et al., 2024; Pulendran and Davis, 2020):

##### 3.2.5.1 Establishment of immune tolerance

Fungi contribute to immune tolerance within the TME by suppressing effector T-cell activity, thereby enabling tumor cells to evade immune surveillance (Arner and Rathmell, 2023; Harris et al., 2024; Hiam-Galvez et al., 2021). Through the modulation of DCs, macrophages, and T-cells, fungi create an immunosuppressive environment that fosters tumor growth (Bilal et al., 2025; Galloway-Peña et al., 2024).

##### 3.2.5.2 Promotion of chronic inflammation

Chronic inflammation driven by fungal infections plays a multifaceted role in TME (Denk and Greten, 2022; Greten and Grivennikov, 2019). Fungi induce the secretion of pro-inflammatory cytokines, including IL-17, IL-6, and TNF-α, which activate immune-suppressive mechanisms that, paradoxically, support tumor survival and progression (Elinav et al., 2013; Lochhead et al., 2021).

### 3.3 Fungi regulate TME through metabolites

The regulatory role of fungal metabolites within TME is a complex and rapidly evolving area of research (Hanus et al., 2021; Xiao et al., 2024). Emerging evidence suggests that fungi influence tumor dynamics not only as pathogenic agents but also by directly or indirectly modulating the TME through their metabolites (Cheng et al., 2024; Saftien et al., 2023). These metabolites contribute to key processes such as tumor initiation, progression, immune evasion, and metastasis (Eniafe and Jiang, 2021; Li et al., 2024a).

#### 3.3.1 Mechanisms of fungal metabolite regulation in the TME

**Figure 4:**
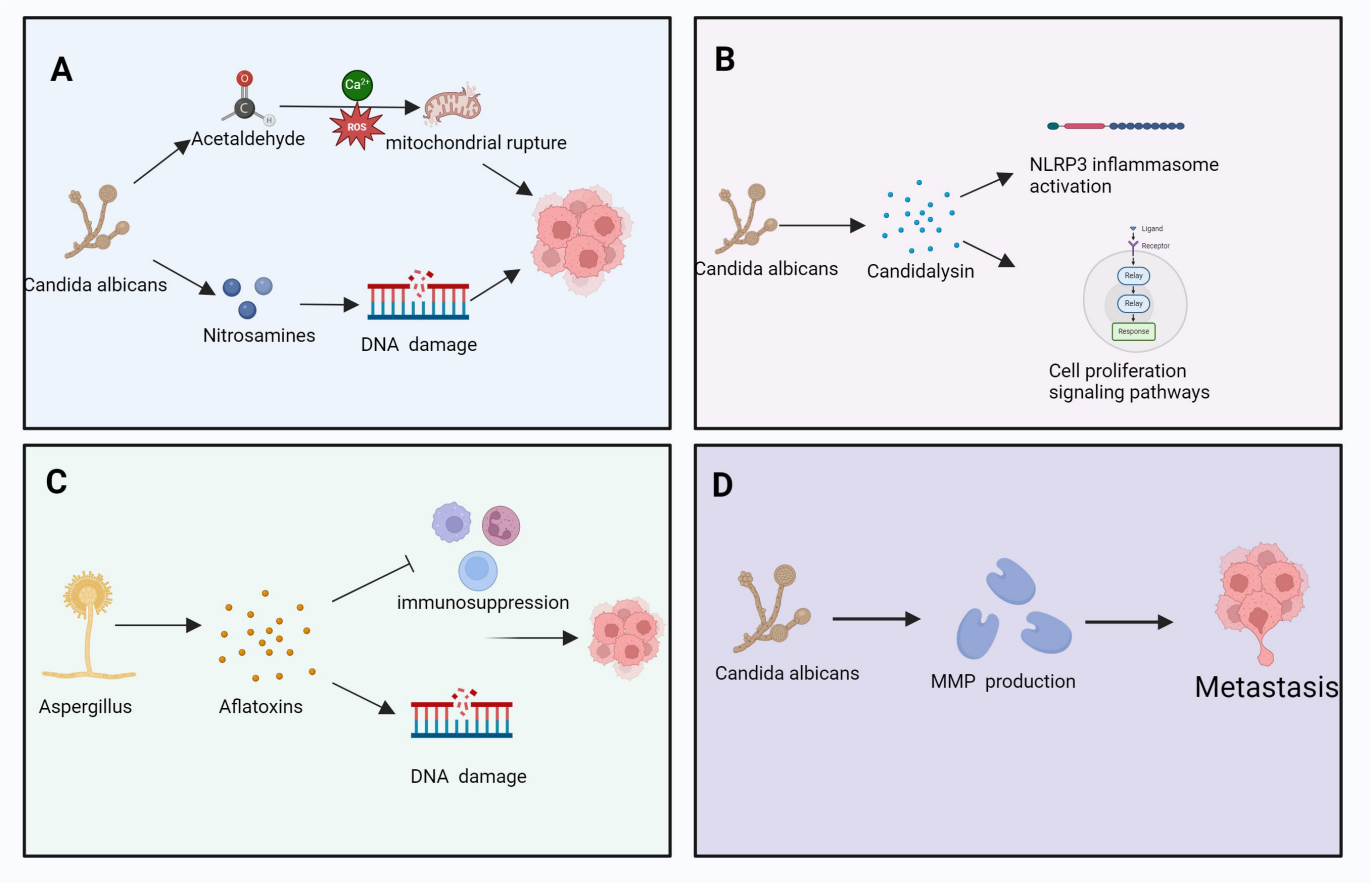
The role of various fungal metabolites in cancer development, involving processes such as DNA damage, immune suppression, cell proliferation, and metastasis. Specifically, it includes: **(A)** *C albicans* induces DNA damage through its metabolites acetaldehyde and nitrosamines. Acetaldehyde generates reactive oxygen species (ROS) mediated by calcium ions (Ca²^+^), leading to mitochondrial rupture, which further disrupts cell function and promotes cancer progression. **(B)** *C albicans* secretes candidalysin, which activates the NLRP3 inflammasome. This process regulates cell proliferation-signaling pathways, promoting tumor cell proliferation and advancing cancer progression. **(C)** *Aspergillus* secretes aflatoxins, leading to immune suppression and DNA damage. The immunosuppressive effect of aflatoxins creates a favorable environment for tumor cell proliferation and survival. **(D)** *C albicans* promotes the production of matrix metalloproteinases (MMPs) through its metabolites, thereby facilitating the metastasis of tumor cells. This process helps tumor cells traverse the basement membrane and spread to other tissues. Pathways with dashed arrows represent hypothetical interactions yet to be validated in clinical studies (Kozieł et al., 2021; Rushing and Selim, 2019).

Fungal metabolites, including mycotoxins, volatile organic compounds (VOCs), and small molecular metabolites, modulate immune responses within the TME (Kozieł et al., 2021; Rushing and Selim, 2019). These compounds can alter immune cell activation, differentiation, proliferation, and responsiveness to tumor cells, thereby influencing immune surveillance and tumor immune evasion (Mafe and Büsselberg, 2024; Pitt and Miller, 2017) (**Figure 4**).

##### 3.3.1.1 Immunosuppressive effects of mycotoxins

Mycotoxins, such as aflatoxin and muscarine, Aflatoxins are produced primarily by Aspergillus flavus and A. parasiticus, while muscarine is associated with Inocybe and Clitocybe species,inhibit immune cell functions, including those of T-cells and macrophages, thereby weakening the host’s antitumor response (Claeys et al., 2020; Marchese et al., 2018; Unicsovics et al., 2024). By altering the immune cell composition within the TME, mycotoxins contribute to a suppressed antitumor immune landscape, enhancing the tumor’s ability to evade immune detection and destruction (Kraft et al., 2021) (**Figure 4A**).

##### 3.3.1.2 Regulation of immune cell metabolism

Fungal metabolites significantly affect immune cell metabolism within the TME, Notable fungal metabolites involved include candidalysin, gliotoxin, and patulin, which modulate immune signaling and epithelial integrity, thereby influencing their functional capacity (Cheng et al., 2024; Wang et al., 2024c; Zhou et al., 2024e). For instance, these metabolites may suppress T-cell effector function by modulating key metabolic pathways, including glycolysis and fatty id oxidation (He et al., 2021; Jia et al., 2024a). In addition, fungal metabolites may promote the accumulation and activation of immunosuppressive cells, such as Tregs and tumor-associated macrophages, further enhancing immune evasion (Campbell et al., 2020; Ma et al., 2017) (**Figures 4B, C**).

#### 3.3.2 Metabolic reprogramming and tumor growth

Fungal metabolites play a role in tumor cell metabolic reprogramming (Baixauli et al., 2022). Cancer cells frequently exhibit the “Warburg effect,” favoring anaerobic glycolysis over oxidative phosphorylation, even in oxygen-rich environments, metabolites such as ethanol, acetaldehyde, and farnesol have been shown to promote glycolysis in tumor and immune cells, so as to meet their energy demands (Takeuchi et al., 2024). So as to meet their energy demands (Takeuchi et al., 2024). Certain fungal metabolites, such as organic acids, ketones, and fatty acids, can influence these metabolic pathways, thereby affecting tumor cell growth and proliferation (Takeuchi et al., 2024). For example, some fungal metabolites enhance glycolysis in tumor cells, thereby providing additional energy to support the rapid tumor expansion (Bacigalupa et al., 2024; Zhou et al., 2024d) (**Figure 4D**).

#### 3.3.3 Regulation of autophagy and apoptosis in tumor cells

Some fungal metabolites influence key cellular processes, including autophagy and apoptosis, within tumor cells. Autophagy is a critical survival mechanism that enables tumor cells to adapt to nutrient deprivation and cellular stress (Bacigalupa et al., 2024). Certain fungal metabolites regulate autophagy, allowing tumor cells to survive unfavorable conditions by modulating pathways such as the mechanistic target of rapamycin signaling (Bacigalupa et al., 2024; Ngwa et al., 2019; Zhou et al., 2024c). Secondary metabolites of fungi may enhance tumor cell survival by interacting with these pathways, thereby contributing to tumor progression and resistance to therapeutic interventions (Lee et al., 2024) (Claeys et al., 2020).

#### 3.3.4 Fungal metabolites and immune cell interactions in the TME

Fungal metabolites significantly affect interactions between immune cells and tumor cells within the TME, reshaping its immune landscape (Takeuchi et al., 2024; Wu et al., 2020). These metabolites can enhance or inhibit the recruitment and infiltration of specific immune cell populations, thereby altering immune cell composition and functionality (Bachem et al., 2019; Chen et al., 2021; Takeuchi et al., 2024). Such changes influence immune evasion mechanisms and tumor growth dynamics (Wu et al., 2024b). Examples include gliotoxin, which suppresses NF-κB activation, and indole-3-lactic acid, which modulates host inflammation through AhR signaling. For example, certain metabolites may increase the infiltration of immunosuppressive cells or decrease the presence of cytotoxic immune cells, tipping the balance in favor of tumor survival (Bacigalupa et al., 2024; Staudt et al., 2023; Uribe-Herranz et al., 2020; Wu et al., 2024b).

#### 3.3.5 Promotion of an immunosuppressive microenvironment

Fungal metabolites play a crucial role in the polarization of tumor-associated macrophages within TME (Rangel Rivera et al., 2021). They facilitate the shift from the pro-inflammatory M1 phenotype, which exerts antitumor effects, to the immunosuppressive M2 phenotype (Wu et al., 2020). This transition enhances immune suppression and creates a microenvironment conducive to tumor growth and immune evasion, allowing cancer cells to proliferate unchecked (Arpaia et al., 2013).

#### 3.3.6 Regulation of cytokine and chemokine expression

The expression of cytokines and chemokines within the TME is intricately regulated by fungal metabolites (Bhat et al., 2022; Propper and Balkwill, 2022). These metabolites modulate immune cell activity by upregulating or downregulating cytokine and chemokine levels (Gupta et al., 2022). For instance, certain fungal compounds may induce tumor cells to secrete immunosuppressive cytokines, such as IL-10 and TGF-β (Gupta et al., 2022; Singha et al., 2015). These cytokines suppress immune responses, reduce the activity of cytotoxic T-cells, and promote Treg function, collectively facilitating tumor immune evasion (Cheng et al., 2016; Drouillard et al., 2023).

#### 3.3.7 Fungal interactions with microbial communities in the TME

The TME encompasses a diverse array of microbial communities, including both bacteria and fungi (Jiang et al., 2024). Fungal metabolites examples include gliotoxin, which suppresses NF-κB activation, and indole-3-lactic acidcan interact with bacterial populations.Fungal metabolites can interact with bacterial populations, influencing the overall microbiota composition and the activity within TME (Jiang et al., 2022a, 2024; Liu et al., 2024a). These interactions may alter immune responses and contribute to tumor progression (Qiu et al., 2020). Recent studies have highlighted the dynamic interplay between fungi and bacteria in modulating tumor growth, immune suppression, and the overall immune milieu (Bi et al., 2024; Qian et al., 2024; Qiu et al., 2020).

#### 3.3.8 Synergistic effects of gut microbiota and immunotherapy

The gut microbiota plays a pivotal role in determining the effectiveness of cancer immunotherapy (Kim and Lee, 2021). Emerging research suggests that fungal communities within the gut microbiota significantly impact immune modulation and therapeutic outcomes (Kim and Lee, 2021; Masheghati et al., 2024; Yao et al., 2024). Certain fungal metabolites influence the composition and functionality of the gut microbiota, thereby affecting the host’s systemic immune responses (Dong et al., 2021; Gao et al., 2021). By shaping the gut microbiota, fungi indirectly modulate the immune characteristics of the TME, potentially enhancing or diminishing the efficacy of immunotherapeutic strategies (Dong et al., 2021; Jamal et al., 2023). These findings underscore the importance of integrating microbiome studies into cancer treatment paradigms so as to optimize therapeutic responses (Zhao et al., 2024).

In 2015, Thomas and his colleagues first noticed that there were correlations between gut microbiota and ICI immunotherapy. They used mice which were harbored with different commensal microbiota, then compared the melanoma growth of these mice. They also found that different microbiota might relate to different spontaneous antitumor immunity. Of which, they found that *Bifidobacterium* could facilitate antitumor effect of PD-L1 blockade (Sivan et al., 2015).

#### 3.3.9 Typical fungal metabolites and their effects on TME

##### 3.3.9.1 Mycotoxins

Aflatoxin, a potent carcinogenic mycotoxin produced by *A. flavus*, directly interacts with DNA, thereby inducing tumor formation (Marchese et al., 2018). In addition to its genotoxic effects, aflatoxin modulates immune system function, promoting tumor cell growth and metastasis by impairing immune surveillance mechanisms and facilitating immune evasion (Dong et al., 2022; Zhu et al., 2021).

##### 3.3.9.2 Ochratoxin

Ochratoxin, secreted by *Aspergillus ochraceus*, exerts significant immunosuppressive effects within the TME (Llobregat et al., 2022; Parussolo et al., 2019). It alters immune cell infiltration and functionality, creating an immune-suppressive milieu that supports tumor progression (Liu et al., 2024d; Więckowska et al., 2024). This mycotoxin disrupts the balance of immune responses, further promoting tumor proliferation and metastatic potential (Liu et al., 2024d; Więckowska et al., 2024).

##### 3.3.9.3 VOCs

Fungal VOCs are small molecules with diverse biological activities, including the regulation of plant growth and modulation of tumor cell behavior (Gouzerh et al., 2022; Zhou et al., 2024b). These VOCs can influence the TME by altering immune cell functions, by either promoting or suppressing immune responses (Gouzerh et al., 2022; Stone, 2022; Zhou et al., 2024b). Through such mechanisms, fungal VOCs indirectly affect immune cell activation, differentiation, and cytokine production, thereby modulating the immune status of the TME and contributing to tumor progression (Gouzerh et al., 2022; Wekking et al., 2024).

##### 3.3.9.4 Secondary metabolites of fungi

Fungal secondary metabolites, such as mycophenolic acid, tacrolimus (cyclosporine), and polyamide compounds, are critical mediators in the TME (Liu et al., 2024b; Nesic et al., 2014). These metabolites can influence tumor immune evasion and cell proliferation (Domingos et al., 2022; Duizer and de Zoete, 2023; Lin et al., 2021; Staszczak, 2021) (He et al., 2020). For instance, tacrolimus, an immunosuppressant commonly used in clinical settings, inhibits T-cell function and contributes to immune suppression, which facilitates tumor cell survival and growth (He et al., 2020) (Julianti et al., 2022).

Fungal interactions within the TME extend beyond direct effects on immune and tumor cells. Metabolites influence microbial communities, regulate immune cell activity, and alter tumor cell metabolism, collectively contributing to immune evasion, proliferation, metastasis, and therapeutic resistance. This complex interplay highlights fungal metabolites as promising targets for innovative antitumor strategies and reinforces the importance of this emerging research area in the field of cancer biology.

### 3.4 Fungi influence TME through angiogenesis and tumor metastasis

#### 3.4.1 Promotion of angiogenesis

Angiogenesis is essential for tumor growth and metastasis, providing tumor cells with oxygen and nutrients while offering a pathway for dissemination (Dudley and Griffioen, 2023b; Wang et al., 2015). Fungal metabolites and structural components, such as β-glucan, play critical roles in promoting angiogenesis by stimulating the release of pro-angiogenic factors, including vascular endothelial growth factor (VEGF) (Choi et al., 2022; Xu et al., 2024). Proposed pathway based on limited experimental evidence; quantitative validation is needed (Xu et al., 2024).

Several fungal species, such as *Aspergillus* and *C. albicans*, induce localized inflammatory responses that enhance angiogenesis through the secretion of metabolites like lipids and toxins (Wang et al., 2024b, 2015). These factors influence vascular remodeling and blood vessel formation within the TME, enabling tumor expansion and metastatic spread (Cao et al., 2023b; Dudley and Griffioen, 2023a, 2023b).

#### 3.4.2 Secretion of angiogenesis factors

Fungal infections activate host immune cells, including macrophages and DCs, prompting the secretion of VEGF and basic fibroblast growth factor (Kuo et al., 2018; Onyishi et al., 2023). These angiogenesis factors drive the formation of new vasculature, supporting tumor growth and facilitating tumor cell migration into the circulatory system (Liu et al., 2023d; Patel et al., 2023).

For example, *Aspergillus* species promote angiogenesis by inducing VEGF production, enhancing nutrient delivery to tumor cells while providing a conduit for metastasis (Ben-Ami et al., 2013; Ito, 2013; Park et al., 2019). Similarly, *C. albicans* and *Cryptococcus* leverage cell wall polysaccharides, such as β-glucan, to activate local immune responses (Iyer et al., 2021). This activation leads to upregulated angiogenesis factor secretion, which further contributes to vascular proliferation and metastatic progression within the TME (Bhat et al., 2021; Pérez-Tomás and Pérez-Guillén, 2020).

#### 3.4.3 Tumor cell metastasis

Fungi contribute to tumor metastasis by altering immune cell composition in the TME, modulating immune evasion mechanisms, and regulating pro-inflammatory cytokines (Arner and Rathmell, 2023; Liu et al., 2024b). Gut microbiota dysbiosis, including fungal imbalances, is closely linked to metastatic progression (Li et al., 2019; Liu et al., 2024b).

Tumor metastasis refers to the dissemination of tumor cells from their primary site to distant tissues—a process facilitated by angiogenesis, immune evasion, and enhanced tumor cell invasiveness (Li et al., 2019; Liu et al., 2024b). Fungi influence metastasis through multiple mechanisms, which significantly alters the TME (Fu et al., 2023).

#### 3.4.4 Regulation of Treg cells and immune evasion

Fungi can induce the accumulation of Tregs, which suppress antitumor immunity (Liu et al., 2024b). Tregs secrete immunosuppressive cytokines such as IL-10 and TGF-β, inhibiting effector T-cell activity and enabling tumor cells to escape immune surveillance (Fu et al., 2023; Liu et al., 2024b). This immune-suppressive environment promotes metastasis, particularly within newly established tumor sites, where conditions favor tumor cell proliferation and spread (Hoshino et al., 2015; Wong and Yu, 2024).

#### 3.4.5 Relationship between angiogenesis and tumor metastasis

Angiogenesis supplies tumor cells with oxygen and nutrients while providing direct routes for tumor cells to enter the circulatory and lymphatic systems(C. Jiang et al., 2021; Singhal and Augustin, 2020). Fungi contribute to angiogenesis through metabolites that enhance endothelial cell proliferation and vascular permeability, thereby increasing tumor cell invasiveness and metastatic potential (Mehrian-Shai et al., 2019; Ribatti, 2024).

#### 3.4.6 Fungal metabolites and the expression of metastasis-related proteins

Fungal metabolites activate signaling pathways associated with metastasis in tumor cells (Sharma-Walia et al., 2012). For example, *C. albicans* infection stimulates the MAPK/ERK pathway, enhancing tumor cell invasiveness and migratory capacity (Ho et al., 2019; Song et al., 2022). This signaling cascade promotes the dissemination of tumor cells to secondary tissues, facilitating metastasis (Sharma-Walia et al., 2012).

#### 3.4.7 Pro-inflammatory factors and tumor metastasis

Chronic inflammation induced by fungal infections promotes the release of pro-inflammatory cytokines, which then contribute to tumor cell migration and metastatic progression (Yaniv et al., 2024; Zhu et al., 2024). Pro-inflammatory mediators such as TNF-α, IL-1β, and IL-6 not only stimulate angiogenesis but also alter tumor cell adhesion and infiltration, thereby enhancing metastatic behavior (Cruceriu et al., 2020; Kobelt et al., 2020; Zhang et al., 2021).

##### 3.4.7.1 Fungi and IL-17 regulation in metastasis

Fungi, including *C. albicans*, drive IL-17 production, a cytokine associated with chronic inflammation and immune suppression (Aggor et al., 2020; Shao et al., 2019). IL-17 fosters an immune-suppressive environment while upregulating pro-inflammatory mediators, thereby increasing tumor cell invasiveness and metastatic potential (Guo et al., 2022; Li et al., 2024b). This dual role makes IL-17 a pivotal link between chronic fungal infections and tumor metastasis (Chen et al., 2023b; Liu et al., 2024c).

### 3.5 Interactions between fungi and symbiotic microbiota in the TME

**Figure 5:**
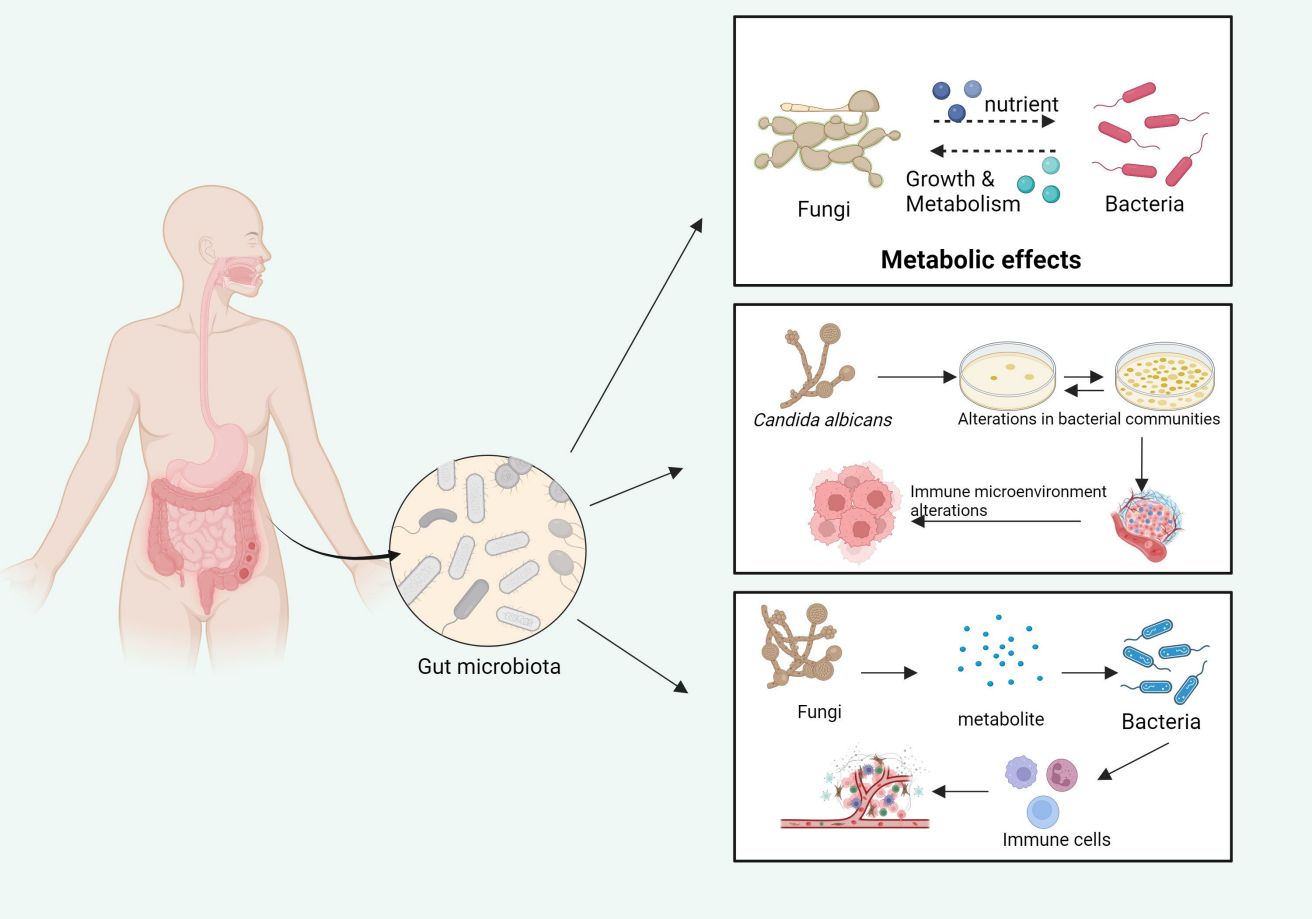
The influence of fungi and bacteria on each other’s growth and metabolism in the gut microbiota. Fungi, by metabolizing various nutrients, impact bacterial growth and metabolic processes. In turn, the metabolites produced by bacteria influence fungal growth and activity. These interactions cause alterations in the gut microbiome, which, in turn, modulate the local immune microenvironment, potentially affecting systemic immune responses (Top panel): fungi, through their metabolic consumption of nutrients, directly influence the growth and metabolic activities of bacterial communities within the gut. This metabolic interaction contributes to the formation of distinct microbial community structures. (Middle panel): Specifically, *C. albicans* can alter the composition of bacterial populations. The resulting disruption in the microbiome leads to significant changes in the immune microenvironment, affecting the host’s immune responses, including the modulation of inflammatory and anti-inflammatory pathways. (Bottom panel): The metabolites produced by fungi interact with bacterial metabolites to influence immune cell activation. These interactions not only alter the gut microenvironment but can also affect systemic immune functions, potentially influencing host susceptibility to infections and disease progression. Pathways with dashed arrows represent hypothetical interactions yet to be validated in clinical studies (Takeuchi et al., 2024).

The complex interplay between fungi and other symbiotic microorganisms within the TME has emerged as a significant area of research (Takeuchi et al., 2024). Fungal metabolites influence the TME directly and indirectly by interacting with bacterial, viral, and other microbial populations (Takeuchi et al., 2024). The collective activity of these microbial communities, particularly the gut microbiota, plays a critical role in tumor initiation, progression, immune evasion, and response to therapy (Gagnière et al., 2016; Pickard et al., 2017). As the key members of the microbiota, fungi contribute to tumorigenesis by modulating the composition and functionality of these microbial networks, thereby reshaping the TME to support tumor growth and metastasis (Heidari et al., 2024; Wang et al., 2024c) (**Figure 5**).

#### 3.5.1 Synergistic effects of fungi and symbiotic microbiota in the TME

The TME comprises not only tumor and immune cells but also diverse microbial communities, including bacteria, fungi, and viruses (Brennan and Garrett, 2019; Schwabe and Jobin, 2013). These microbial populations exert significant influence on tumor biology through intricate interactions (Schwabe and Jobin, 2013). As integral members of these communities, fungi interact with symbiotic microbiota in ways that shape the TME (Chen et al., 2017).

##### 3.5.1.1 Interactions among microbial communities

Fungi in the TME do not exist in isolation; they interact dynamically with bacteria, viruses, and other microorganisms (Chen et al., 2017; Wang et al., 2024c). For instance, certain fungal metabolites may serve as nutrients for bacteria or modify microbial metabolic outputs, notably, Bacteroides, Lactobacillus, and Prevotella species appear to benefit from fungal metabolic interactions, thereby influencing the overall microbiota composition (Wang et al., 2024c). Conversely, bacterial metabolic by-products, such as short-chain fatty acids and lactic acid, can impact fungal growth and metabolism, thereby creating bidirectional regulatory networks that affect TME (Koliarakis et al., 2019; Lu et al., 2022).

##### 3.5.1.2 Impact of gut microbiota on immunotherapy

A growing body of research has highlighted the critical role of gut microbiota composition in determining the success of cancer immunotherapy (Galloway-Peña et al., 2024; Wong and Yu, 2023). Certain gut bacteria enhance antitumor immune responses, and fungi may act as modulators in this process (Dohlman et al., 2021; Tong et al., 2021). Fungal metabolites can influence bacterial populations within the gut, alter immune responses, and subsequently affect tumor immune evasion and therapeutic outcomes (Hanus et al., 2021; Li et al., 2023a; Wang et al., 2024c).

#### 3.5.2 Fungi and gut microbiota interactions

The gut microbiota, comprising bacteria, fungi, viruses, and other microorganisms, plays a pivotal role in host health, immune regulation, and disease progression (Li et al., 2023a, 2024a; Papon et al., 2021; Wang et al., 2024c). Dysbiosis within this complex ecosystem has been closely linked to cancer development, with fungi contributing to gut microbiota alterations and tumor progression through several mechanisms (Li et al., 2023b; Wang et al., 2024c).

##### 3.5.2.1 Fungi’s role in maintaining gut microbial balance

Fungal species such as *C. albicans* and *Aspergillus* are the normal components of the gut microbiota, where they help maintain microbial homeostasis (Li et al., 2023b; Wang et al., 2024c). However, conditions such as immunosuppression or antibiotic use can disrupt this balance, leading to fungal overgrowth and dysbiosis (Gou et al., 2024; Shi et al., 2020). Fungal dysbiosis can destabilize the gut microbiota equilibrium, fostering an environment conducive to tumor initiation and progression (Bi et al., 2024; Chen et al., 2024a; Malik et al., 2018). Furthermore, fungal metabolites can reshape the bacterial community, activating immune cells and modulating antitumor immune responses, either enhancing or suppressing immune activity (Bi et al., 2024).

##### 3.5.2.2 Relationship between gut microbiota and immune response

The gut microbiota profoundly influences local and systemic immune responses, thereby directly impacting tumor immune evasion (Guo et al., 2021; Schürch et al., 2020; Zhou et al., 2021). Bacterial metabolic products, such as short-chain fatty acids, enhance local immune activity and suppress tumor growth (Chen et al., 2022; Jiang et al., 2023). Fungi indirectly affect immune regulation by modifying the synthesis of these metabolites (Chen et al., 2022; Jiang et al., 2023; Ngwa et al., 2019; Wang et al., 2023a). For example, *C. albicans* interacts with gut bacteria to either stimulate or suppress immune responses, thereby playing a pivotal role in tumor initiation and progression through its influence on immune modulation and microbiota composition (d’Enfert et al., 2021; Pierre et al., 2023).

##### 3.5.2.3 Immunotherapy and microbiota interactions

Increasing evidence suggests that the composition of the gut microbiota significantly influences the effectiveness of immunotherapy (Routy et al., 2018; Trompette et al., 2014). For example, the efficacy of immune checkpoint inhibitors, such as PD-1/PD-L1 inhibitors, is closely linked to the gut microbiota (Routy et al., 2018). The fungal communities within the gut may play a role in this response (Trompette et al., 2014). Certain fungal metabolites, through interactions with the gut bacteria, can either enhance or suppress the effectiveness of these inhibitors (Matson et al., 2021). Therefore, modulating the gut microbiota, particularly by balancing the relationship between fungi and bacteria, may offer a novel strategy to improve immunotherapy outcomes (Chrysostomou et al., 2023; Matson et al., 2021; Routy et al., 2018; Zhou et al., 2021).

While fungal modulation of the gut microbiota may impact immunotherapeutic efficacy, findings remain inconsistent across studies. Some reports failed to demonstrate a significant correlation between fungal diversity and immune checkpoint response, potentially due to confounding factors such as antibiotic exposure, diet, tumor type, and baseline immune heterogeneity. Therefore, a clearer understanding of how fungi interact with host immunity—and under what circumstances they enhance or suppress therapy—is still required (Chrysostomou et al., 2023).

#### 3.5.3 Fungi and interactions with other microbial communities

Beyond the gut microbiota, fungi also interact with other microbial populations within the TME, such as the skin and oral microbiota, thereby influencing tumor initiation and progression (Flowers and Grice, 2020; Hong et al., 2019).

##### 3.5.3.1 Oral microbiota and tumors

Fungi in the oral cavity, especially *Candida*, are strongly associated with the development of oral and esophageal cancers (Alnuaimi et al., 2015; Deshpande et al., 2018). *Candida* interacts with bacterial communities in the oral cavity through its metabolites, altering the local immune environment and promoting tumor growth (Huo et al., 2022; Shuai et al., 2022). Certain fungi may directly stimulate tumor cells or modulate local immune responses, thereby contributing to tumor progression and metastasis (Wang et al., 2024c).

##### 3.5.3.2 Skin microbiota and tumors

Fungi on the skin, such as *Candida* and *Malassezia*, are vital for maintaining the skin microbiota balance (Hau et al., 2015). The fungal community on the skin is linked to the development of skin cancers, including melanoma (Hanes et al., 2021; Shiao et al., 2021). Fungi may promote tumor development by influencing the local immune response or by interacting with skin bacteria, thereby further contributing to tumor initiation and progression (Byrd et al., 2018; Li et al., 2023c).

#### 3.5.4 Therapeutic potential of modulating the TME

Modulating fungal communities or balancing their metabolites within the TME may provide new avenues for cancer treatment (Narunsky-Haziza et al., 2022). Adjusting the fungal populations in the gut or other microbiota may enhance tumor immune responses and improve the efficacy of antitumor immunotherapy (Narunsky-Haziza et al., 2022). In addition, natural products derived from fungi, such as antifungal drugs (e.g., voriconazole) or their metabolites, may serve as adjunctive agents in cancer therapy (Bilal et al., 2025; Dickson, 2019).

Fungi interact with symbiotic microbiota within the TME, including gut, oral, and skin microbiota, influencing tumor initiation, progression, immune evasion, and treatment response. Through their metabolites—such as mycotoxins and VOCs—fungi modulate immune systems, metabolic pathways, and tumor cell behavior. Investigating the mechanisms of fungal interactions with the microbiota in the TME offers valuable insights for tumor immunotherapy and microbiota modulation, potentially leading to breakthroughs in future cancer treatments.

### 3.6 Antifungal therapy and cancer treatment

**Figure 6:**
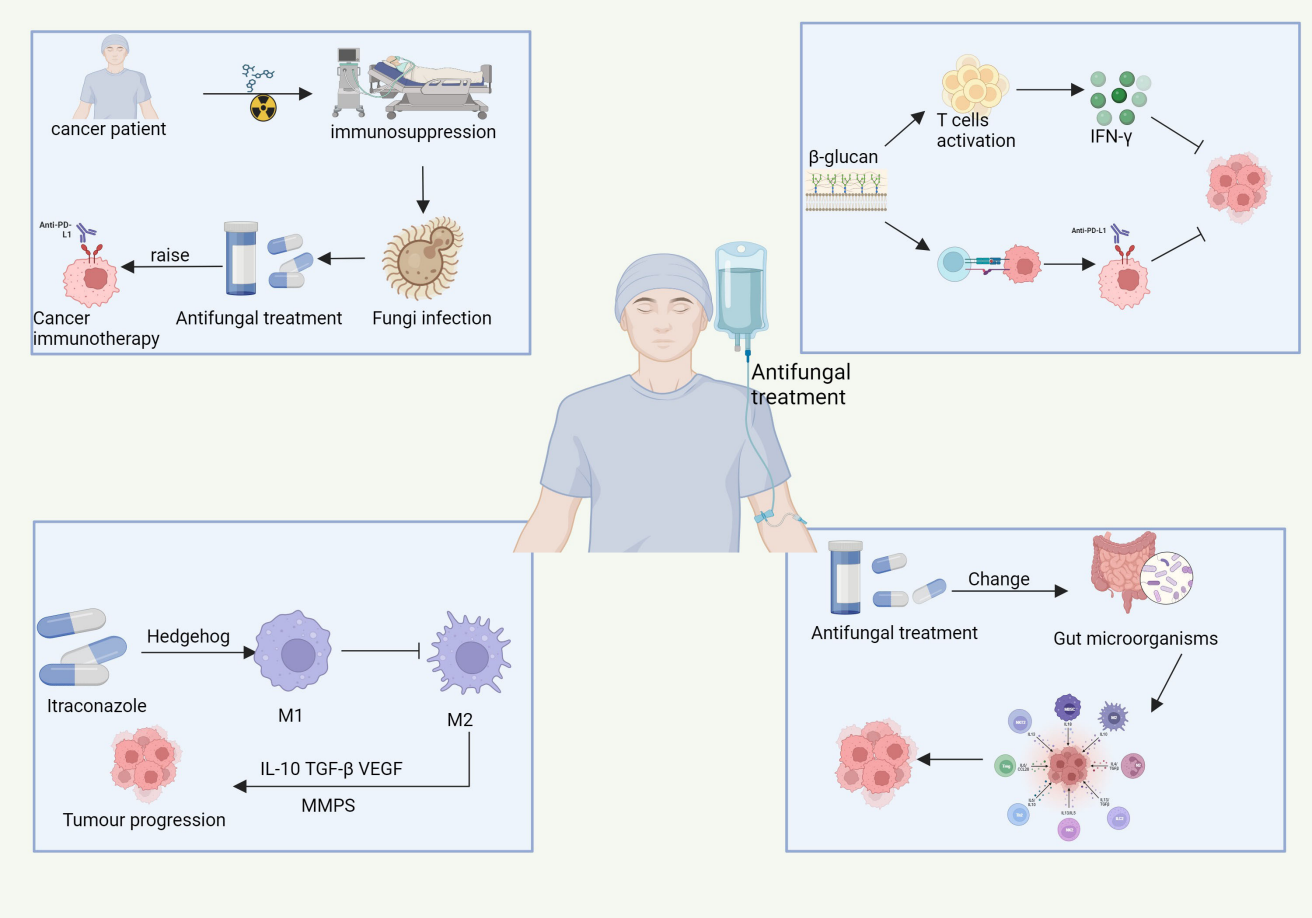
The complex interactions between antifungal treatment, cancer immunotherapy, and tumor progression in immunocompromised cancer patients. Cancer therapies, such as chemotherapy and immunotherapy, induce immune suppression, increasing vulnerability to fungal infections. These infections can complicate cancer treatment. Antifungal treatments, like β-glucan, not only help combat fungal infections but also activate immune responses, stimulating T-cells and promoting IFN-γ production. This immune activation can enhance the effectiveness of cancer immunotherapy, particularly anti-PD-L1 therapy. Furthermore, antifungal treatments such as itraconazole influence tumor progression by modulating immune pathways, including the Hedgehog-signaling pathway, which shifts macrophage polarization from the immune-activating M1 phenotype to the immunosuppressive M2 phenotype, supporting tumor growth. Moreover, antifungal treatment can alter the gut microbiota, indirectly influencing systemic immunity and affecting cancer progression. Pathways with dashed arrows represent hypothetical interactions yet to be validated in clinical studies (Jia et al., 2024b; Yang et al., 2022a).

The integration of antifungal therapy with cancer treatment is an emerging and promising area of research (Bilal et al., 2025; Weng et al., 2023). While much of the current literature focuses on the interactions between fungal infections and the immune systems of patients with cancer, an increasing body of evidence suggests that antifungal therapy can play a more significant role than merely addressing infections (Liu et al., 2023b; Neoh et al., 2024; Weng et al., 2023). In fact, antifungal therapy may have the potential to regulate the TME and improve cancer treatment outcomes (Jia et al., 2024b; Yang et al., 2022a) (**Figure 6**).

#### 3.6.1 Impact of antifungal therapy on the immune system

Cancer therapies such as chemotherapy, radiotherapy, and immunotherapy often induce immunosuppression, rendering patients with cancer more susceptible to fungal infections (e.g., *Candida* and *Aspergillus*) (K. Li et al., 2021). Under these circumstances, apart from eliminating infections, antifungal therapy may have important effects on immune system modulation (Lionakis et al., 2023; Pathakumari et al., 2020).

##### 3.6.1.1 Restoration of immune function

Immunosuppression is a significant driver of tumor immune evasion(X. Cao et al., 2023a; Chen et al., 2023). By clearing fungal infections, antifungal drugs can help restore immune function, alleviate immune system stress, and promote more robust antitumor immune responses (Fisher et al., 2022; Iyer et al., 2021). For example, antifungal drugs can inhibit the growth of immunosuppressive fungi, such as *Candida*, thereby preventing them from dampening immune responses and potentially enhancing the efficacy of cancer immunotherapy (Lionakis et al., 2023; H. Lu et al., 2023).

##### 3.6.1.2 Modulation of the immune microenvironment

Certain antifungal drugs—such as voriconazole and itraconazole—may modulate the immune cell landscape within the TME (Jang et al., 2023; Jia et al., 2024b). These drugs can influence immune cell polarization, leading to enhanced antitumor immune responses (Jia et al., 2024b; Yang et al., 2022a). For instance, antifungal therapy stimulates DCs, which play a pivotal role in antigen presentation. Enhanced DC activity leads to improved T-cell activation, which can intensify the body’s immune response against the tumor (Fites et al., 2018; Wang et al., 2021).

#### 3.6.2 Direct effects of antifungal drugs on tumor cells

In addition to their immune-modulating effects, some antifungal drugs have demonstrated direct antitumor activity (Shanholtzer et al., 2022; Wei et al., 2017). These drugs can influence tumor cell proliferation, migration, and resistance to treatment through a variety of mechanisms (Dembitsky et al., 2021; Yamaguchi et al., 1993).

##### 3.6.2.1 Inhibition of tumor cell proliferation

Antifungal agents such as voriconazole and itraconazole have been identified as having antitumor properties, including the ability to inhibit tumor cell proliferation (Benitez and Carver, 2019; D’Arcy et al., 2020). These drugs exert their effects by disrupting the key metabolic pathways involved in tumor growth, such as fatty acid synthesis, or by targeting signaling pathways such as the mechanistic target of the rapamycin pathway that is critical for tumor cell survival and proliferation (Liu et al., 2023b; Weng et al., 2023).

##### 3.6.2.2 Enhancing drug sensitivity

Antifungal drugs may enhance the sensitivity of tumor cells to chemotherapy or immunotherapy, thereby improving the overall efficacy of these treatments (Weng et al., 2023). For instance, several studies have demonstrated that certain antifungal drugs can potentiate the effects of chemotherapy by inhibiting multidrug resistance proteins (MDR) in tumor cells, thereby reducing tumor cell resistance to chemotherapy agents (Bilal et al., 2025; Weng et al., 2023).

##### 3.6.2.3 Induction of tumor cell apoptosis

Certain antifungal drugs induce apoptosis in tumor cells (Chen et al., 2019). Fungal cell wall components and metabolites can bind to receptors on the surface of tumor cells, triggering apoptotic signaling pathways and leading to programmed cell death (Chakrabarti and Ray, 2016; Yang et al., 2023). This ability to induce apoptosis can further enhance the therapeutic effects of antifungal agents when combined with other cancer treatments (Forma and Bryś, 2021).

#### 3.6.3 Synergistic effects of antifungal therapy and immune checkpoint inhibitors

Immune checkpoint inhibitors (such as PD-1/PD-L1 inhibitors and CTLA-4 inhibitors) have emerged as a significant advancement in cancer immunotherapy (Naimi et al., 2022). Moreover, antifungal therapy may have a synergistic effect when used in conjunction with immune checkpoint inhibitors, potentially improving the outcomes of these treatments (Butterfield and Najjar, 2024; Jia et al., 2024a).

While several studies suggest that gut fungi may enhance immune checkpoint blockade efficacy, other analyses have reported inconsistent associations, possibly due to antibiotic use, diet, or inter-individual variability. Thus, the role of the mycobiome in immunotherapy response remains complex and warrants further investigation.

##### 3.6.3.1 Regulation of microbiota

The gut microbiota plays a crucial role in determining the efficacy of immune checkpoint inhibitors (Li et al., 2022a). Dysbiosis in the gut microbiota can negatively affect the effectiveness of these inhibitors (Derosa et al., 2024). Antifungal drugs, by regulating the fungal populations within the gut microbiota, may help restore microbial balance, which could, in turn, enhance the efficacy of immune checkpoint inhibitors (Wurster et al., 2022). This modulation of the microbiota highlights a novel mechanism through which antifungal therapy can augment the effectiveness of immunotherapy (Mercer and O’Neil, 2020).

##### 3.6.3.2 Modulation of the immune microenvironment

Antifungal therapy may influence the immune cell composition within the TME (Yang et al., 2022a). By modulating the activity of immune cells such as macrophages, DCs, and T-cells, antifungal drugs can promote more efficient tumor antigen presentation and activate immune responses (Jang et al., 2023; Zhuo et al., 2022). For example, certain antifungal drugs, such as voriconazole and itraconazole, may activate specific immune pathways that boost antitumor immune responses (Benitez and Carver, 2019). This effect, in turn, enhances the therapeutic effects of immune checkpoint inhibitors, contributing to improved outcomes in cancer immunotherapy (Jang et al., 2023; Jia et al., 2024b).

#### 3.6.4 Effects of antifungal drugs in the TME

The fungal communities, immune cells, and fibroblasts within the TME interact to influence tumor progression (Aykut et al., 2019). Antifungal therapy not only clears infections and directly affects TME but may also regulate TME indirectly through the following mechanisms:

##### 3.6.4.1 Impact on immune cells in the TME

Interactions between fungi and various immune cells in the TME, such as macrophages and DCs, may contribute to tumor immune evasion (Harris et al., 2024). Antifungal therapy has the potential to restore normal immune cell function and enhance the immune microenvironment within the TME (Jia et al., 2024b; Yang et al., 2022a). This improvement can lead to enhanced antitumor immune responses, thereby supporting the body’s ability to fight the tumor more effectively (Yang et al., 2022a).

##### 3.6.4.2 Alleviating the immunosuppressive microenvironment

Certain fungi, such as *Candida*, can interact with the immune system to create an immunosuppressive environment that promotes tumor growth and metastasis (Lin et al., 2023). By clearing these fungi, antifungal therapy may reduce immune tolerance, thereby strengthening the immune system’s capacity to target and destroy tumor cells (Daley et al., 2017). This process not only improves immune surveillance, but may also inhibit tumor progression.

#### 3.6.5 Effects of antifungal therapy on the gut microbiota

The growing recognition of the role of gut microbiota in tumor immunotherapy has highlighted the potential influence of antifungal drugs on the composition of the gut microbiota (Fernandes et al., 2022; Park et al., 2022; Wong and Yu, 2023). Past studies have suggested that antifungal therapy can alter the structure of the gut microbiota, which, in turn, could modify the host’s immune responses and metabolic state, thereby indirectly impacting tumor growth and immune surveillance (d’Enfert et al., 2021; Renga et al., 2024). For example, certain antifungal drugs, such as amphotericin B, directly target the gut fungal community, which potentially disrupts its balance and subsequently affects both immune responses and antitumor activity (Demir et al., 2022; Wheeler et al., 2016).

#### 3.6.6 Combination of antifungal therapy with radiotherapy/chemotherapy

Antifungal drugs can be integrated into combination therapies with radiotherapy or chemotherapy to improve the treatment outcomes (Souza et al., 2020). The following are the key mechanisms of how antifungal therapy possibly augments these conventional treatments:

##### 3.6.6.1 Enhancing chemotherapy effects

Chemotherapy often leads to immunosuppression, which increases the risk of fungal infections (Wojciechowski and Wiseman, 2021). Antifungal drugs can mitigate this risk, while also potentially enhancing chemotherapy’s antitumor effects by modulating the immune microenvironment (Wojciechowski and Wiseman, 2021). Furthermore, some antifungal drugs possess direct antitumor properties and may synergize with chemotherapy drugs, enhancing their efficacy (Saeedi et al., 2019).

##### 3.6.6.2 Improving radiotherapy effects

Certain antifungal drugs, such as voriconazole, can improve the effects of radiotherapy (Cucchetto et al., 2015). These drugs may help modulate immune cells and tumor cell responses within the TME, thereby enhancing immune cell function and increasing tumor cell sensitivity to radiation (Dandachi et al., 2018). By serving as potential adjuncts to radiotherapy, antifungal drugs can offer an additional means to improve treatment outcomes (Dandachi et al., 2018).

#### 3.6.7 Side Effects and challenges of antifungal therapy

Despite the potential benefits of antifungal drugs in cancer treatment, their use presents several challenges, particularly in terms of the side effects induced during prolonged treatment.

##### 3.6.7.1 Drug Toxicity

Some antifungal drugs may lead to toxicity in the organs such as the liver and kidneys, especially in immunosuppressed patients with cancer (Assress et al., 2020; Silva et al., 2023). This effect necessitates careful monitoring of the organ function throughout antifungal therapy (Silva et al., 2023). Dose adjustments may be required to minimize toxicity and prevent harm, which underscores the importance of a personalized approach to antifungal treatment in patients with cancer (Silva et al., 2023).

##### 3.6.7.2 Drug resistance

Fungi can develop resistance to antifungal drugs, particularly with prolonged use, particularly with prolonged use of specific agents (Fisher et al., 2018). This resistance poses a significant challenge to effective treatment, necessitating the continuous development of new antifungal agents (Perlin et al., 2017). Ongoing research should therefore explore novel antifungal compounds, alternative therapeutic strategies, and approaches to mitigate the emergence of such resistance cases (Perlin et al., 2017).

The combination of antifungal therapy with cancer treatment offers valuable new insights into cancer care. By regulating the TME, restoring immune system function, enhancing immunotherapy efficacy, and directly inhibiting tumor cell proliferation, antifungal drugs could serve as an important adjunct in cancer therapy. Future research is likely to reveal the intricate interactions between fungi and the TME, thereby uncovering the full potential of antifungal drugs in cancer treatment. Meanwhile, ensuring the rational use of antifungal agents and mitigating their potential side effects are deemed crucial in refining future treatment strategies. long-term toxicities of antifungal agents, such as hepatotoxicity or nephrotoxicity, require further investigation in cancer patients (**Table 1**).

**Table 1:** summarizes key antifungal agents with relevance to both fungal control and potential tumor modulation mechanisms.

Antifungal Drugs	Mechanisms	Dose (Typical)	Side Effects	Combination with Chemotherapy/Radiotherapy/Immunotherapy	Referance
Amphotericin B	Direct inhibition of fungal cell wall synthesis, modulation of immune responses, gut microbiota modulation	0.5–1 mg/kg/day	Nephrotoxicity, infusion-related reactions, electrolyte disturbances	May enhance chemotherapy and radiotherapy efficacy, modulate immune system	(Golestannejad et al., 2024; Hiddemann et al., 1991; Petrikkos and Skiada, 2007)
Voriconazole	Inhibition of fungal ergosterol synthesis, immune modulation, activation of dendritic cells	4–6 mg/kg/day	Hepatotoxicity, visual disturbances, GI disturbances	May synergize with chemotherapy, immunotherapy, and modulate immune responses	(Golestannejad et al., 2024) (Hiddemann et al., 1991)
Itraconazole	Inhibition of ergosterol synthesis, immune modulation, inhibition of tumor cell proliferation	200–400 mg/day	Hepatotoxicity, GI disturbances, rash	Enhances chemotherapy sensitivity, modulates immune environment	(Baddley and Pappas, 2005) (Puumala et al., 2024)
Fluconazole	Inhibition of fungal ergosterol synthesis, immune modulation, gut microbiota regulation	400 mg/day	GI disturbances, hepatotoxicity, rash	Enhances chemotherapy and immunotherapy sensitivity, modulates gut microbiota	(Freifeld et al., 2011; Groll and Tragiannidis, 2009; Iyer et al., 2021)
Posaconazole	Inhibition of ergosterol synthesis, immune modulation, inhibition of tumor cell proliferation	300 mg/day	GI disturbances, hepatotoxicity, liver failure	Enhances chemotherapy and immunotherapy efficacy, modulates immune microenvironment	(Puumala et al., 2024; Sable et al., 2008; Sawant and Khan, 2017)
Caspofungin	Inhibition of β-glucan synthesis, modulation of immune responses	50–70 mg/day	Fever, rash, thrombophlebitis	May enhance chemotherapy efficacy, modulate immune response	(Iyer et al., 2021; Petrikkos and Skiada, 2007)
Micafungin	Inhibition of β-glucan synthesis, modulation of immune responses	50–100 mg/day	Fever, rash, thrombophlebitis	Enhances chemotherapy efficacy, modulates immune microenvironment	(Bouz and Doležal, 2021; Freifeld et al., 2011; Groll and Tragiannidis, 2009)

## 4 Summary

The research on the role of fungi in cancer is still in its early stages, with several challenges hindering its progress. One of the main obstacles is the difficulty in detecting fungi within the TME. While fungal detection technologies, such as FungiQuant, have made considerable strides, issues such as sampling difficulties, genomic contamination, operational complexity, and challenges in clinical application remain unresolved. Despite these challenges, the recognition of fungi’s potential role in cancer initiation and progression is growing. However, the mechanisms through which fungi influence cancer remain complex and multifaceted, warranting further investigation. To bridge the gap between basic and clinical research, future studies should focus on deepening our current understanding of the relationship between fungi and cancer, while simultaneously developing more effective strategies for diagnosis, treatment, and prevention.

The relationship between fungi and cancer is a multidimensional research area with considerable promise. Future advancements in this field may include the following: 1. exploration of the impact of fungal infections on cancer initiation and progression, 2. Investigation of the anticancer potential of fungal metabolites and their therapeutic implications, 3. Promotion of fungal-mediated immune modulation in clinical settings, and 4. Identification of novel fungal-related biomarkers for early detection and tailored treatment strategies. Furthermore, the integration of novel antifungal drugs with immunotherapy presents an exciting frontier for future cancer treatment research. As advancements in molecular biology, genetic engineering, immunology, and other related fields continue, the application of fungal resources in cancer care is believed to lead to innovative strategies and groundbreaking approaches for cancer treatment.

## 5 Future directions and priority areas

Moving forward, key priorities include: (1) establishing standardized protocols for mycobiome profiling, (2) validating fungal biomarkers in large multi-cohort studies, (3) dissecting causal versus correlative fungal-tumor interactions using functional models, and (4) exploring the pharmacodynamics and clinical integration of antifungal therapy in oncology. Addressing these gaps will be essential for translating mycobiome insights into clinical applications.
